# Supplementary material for: Minimizing isotropic and deviatoric membrane energy – An unifying formation mechanism of different cellular membrane nanovesicle types
Source: PLoS One. 2020 Dec 31;15(12):e0244796. doi: 10.1371/journal.pone.0244796 (PMC7775103; doi:10.1371/journal.pone.0244796)
Supplement: Attachment — Submitted filename: Response to reviewers.docx [file pone.0244796.s001.docx]

Dear prof. Yi,

thank you for informing us that our manuscript can be considered for publication after a minor revision. We thank the reviewer, especially for thorough reading of the work and pointing to the negligent mistake in one of the equations.

Comment of the reviewer: in eq (7), line 670, there is a typo. There should be (m2/m) instead of (m1/m) in the second lg() of entropy of mixing term

Answer: We have corrected the mistake (previously in line 670 and in the new version in line 707). It is highlighted yellow in the marked version.

Also, we have found 4 typing errors in further reading of the manuscript and have corrected them. We have followed the instructions for preparation of the manuscript form and inserted figure captions at the places where the figures should appear (after the paragraph where they were first mentioned). We have revised the titles of the figures. We have formatted the headings as required. These changes have changed the numbering of the text lines with respect to the previous version. All the corrections are marked yellow.

We hope that the manuscript is appropriate for publication. Also, we hope that this work, if published, will bring something meaningful to the scientific society.

Sincerely yours,

Veronika Kralj-Iglič, in behalf of all authors
